# Digital Affective Resilience: A Cross‐Sectional Observational Study of Anxiety‐Related Chinese Social Media

**DOI:** 10.1002/nop2.70691

**Published:** 2026-07-11

**Authors:** Jinji Chen, Jin Gong, Ziqin Wang, Lili Yang, Yuting Yang, Qiuyi Xu, Shuyan Zhang, Xiaoyan Tian, Lihui Yan, Qi Zhou

**Affiliations:** ^1^ Hangzhou Linping District Integrated Traditional Chinese and Western Medicine Hospital Hangzhou China; ^2^ School of Medicine Sir Run Run Shaw Hospital of Zhejiang University Hangzhou China; ^3^ Zhejiang Shuren University Shulan International Medical College Hangzhou China; ^4^ The Second Clinical College Zhejiang Chinese Medical University Hangzhou China; ^5^ Nursing Department Hangzhou Linping District Integrated Traditional Chinese and Western Medicine Hospital Hangzhou Zhejiang China

**Keywords:** anxiety, emotions, health, media, qualitative study

## Abstract

**Background:**

Anxiety disorders, including phobias, are a growing public health concern, profoundly affecting quality of life. While existing research utilizes text‐based and physiological data for detection, a multimodal, ecologically valid understanding of how anxiety is expressed and regulated in natural social contexts remains limited. Social media offers a unique setting for studying spontaneous emotional disclosure and collective coping mechanisms.

**Methods:**

A text‐mining study was conducted on 28,349 social media comments related to phobia/anxiety from three Chinese platforms (XiaoHongShu, Zhihu, and Weibo) using convenience sampling of publicly available posts up to November 1, 2025. Social media comments related to phobia discussions were collected and analysed using the Dalian University of Technology Chinese Sentiment Vocabulary Ontology lexicon‐based methods. Demographic variables were analysed using Independent Samples t‐test and One‐way ANOVA.

**Results:**

The most frequent emotion categories were happiness (31.6%) and surprise (15.3%), followed by fear (18.4%), sadness (14.7%), anger (12.1%), and disgust (7.9%). Gender differences based on complete‐case analysis (*n* = 12,845) showed that female users expressed significantly more happiness‐ and sadness‐related language than male users (*p* = 0.005 and *p* = 0.034, respectively). The sentiment classifier achieved moderate performance (F1 = 0.72).

**Conclusion:**

The emotional discourse surrounding phobia on Chinese social media reflects co‐occurring linguistic patterns of fear alongside happiness, surprise, sadness, anger, and low‐frequency disgust, rather than fear amplification alone. These findings suggest that online communication may shape how anxiety‐related emotions are collectively expressed and interpreted, although causal inferences cannot be drawn from cross‐sectional text data.

**Implications for the Profession and/or Patient Care:**

This study addressed the limited understanding of anxiety‐related emotional expression on Chinese social media. The findings showed that anxiety discourse involved not only fear, but also supportive, empathetic, and coping‐oriented emotions. These results may help nurses and mental health professionals better understand digital emotional communication, improve psychosocial support, and inform AI‐assisted emotional monitoring and online mental health interventions.

**Reporting Guideline:**

This study was reported in accordance with the STROBE (Strengthening the Reporting of Observational Studies in Epidemiology) Statement for cross‐sectional observational studies.

**Patient or Public Contribution:**

No patients or members of the public were directly involved in the design, conduct, analysis, or manuscript preparation of this study. The research was based on secondary analysis of publicly available and anonymized social media data.

## 1 Background

Anxiety is a psychological and emotional state characterized by excessive worry, anticipatory fear (Crocq 2015; Koskinen and Hovatta 2023), physiological arousal and heightened vigilance toward potential threats (Saeed et al. 2019). Globally and within China, the burden of anxiety disorders has been increasing steadily (Aqtam, Naghnaghiyeh, et al. 2025; Aqtam 2025), with national estimates showing more than a 30% growth in diagnosed anxiety conditions over the past three decades (Crocq 2015; Koskinen and Hovatta 2023). Anxiety profoundly affects social functioning, quality of life and health behaviour, often co‐occurring with sleep disturbance, avoidance behaviours, and emotional dysregulation (Pitman et al. 2018). Because anxiety is fundamentally expressed and communicated through emotions—such as fear, tension, catastrophizing thoughts and negative affect—investigating anxiety‐related emotional patterns is essential for identifying psychological risks and improving early detection strategies (Julian 2011). Understanding how individuals verbalize and negotiate anxiety in real‐world settings can therefore offer unique insights into both the subjective experience and the social meanings of anxiety.

Recent technological advances have brought sentiment analysis—an approach combining natural language processing and machine learning—to the forefront of mental‐health research (Phiri et al. 2025). Text‐based studies have shown that linguistic features, such as negative affective words (Glaz et al. 2021), cognitive processing terms or first‐person pronouns, can prospectively predict changes in depression, generalized anxiety, and social anxiety symptoms. For example, research using 16‐week naturalistic text messages from adults demonstrated that emotional and cognitive language patterns can serve as digital phenotypes for future symptom trajectories. Other studies employing large‐scale online counselling transcripts have identified heterogeneous emotional trajectories—such as stable improvement or fluctuating distress—highlighting that dynamic emotional changes provide more information than single‐time‐point emotional intensity. Social media–based deep‐learning analyses during public health crises have revealed that collective fear and sadness rise sharply during disease outbreaks and correlate with objective epidemiological indicators. Although these studies show that text‐based sentiment analysis is evolving from simple emotional labeling to dynamic prediction and event‐linked modelling, most research focuses on depression, generalized anxiety or broad negative affect. In the context of Chinese social media, evidence regarding how six discrete emotions (fear, sadness, happiness, anger, surprise, and disgust) co‐occur within phobia‐related discourse remains limited and fragmented. Most existing sentiment analysis studies on mental health focus on depression or generalized anxiety, leaving phobia‐specific emotional patterns largely unexplored.

Physiological‐signal approaches, such as electroencephalography (EEG), have been widely used to objectively assess anxiety and fear responses (Glaz et al. 2021; Russo et al. 2024). Machine‐learning models based on neural connectivity and virtual reality exposure paradigms have demonstrated high classification accuracy in identifying anxiety severity and specific phobias (Apicella et al. 2025). However, these approaches primarily capture neurophysiological responses under controlled conditions and do not incorporate individuals' subjective narratives or everyday emotional expression. The present study does not use physiological data; instead, it leverages social media as an ecologically valid source of naturalistic emotional expression and applies sentiment analysis to examine how anxiety is communicated and socially interpreted in real‐world contexts.

Social media provides an important opportunity to fill this gap: its anonymity, openness and real‐time interaction facilitate spontaneous, less inhibited emotional disclosure that is often absent in clinical or survey‐based assessments (Song et al. 2024). At the same time, misinformation, emotional amplification and selective self‐presentation pose analytical challenges, underscoring the need for machine‐learning methods combined with qualitative interpretation to uncover authentic emotional meanings. Therefore, the present study uses sentiment analysis supported by machine learning to examine anxiety‐related emotional expressions on Chinese social media. By integrating computational classification with qualitative, context‐sensitive interpretation, this study aims to reveal dominant emotional patterns, the social construction of anxiety discourse and the digital dynamics of collective affect, thereby contributing methodological innovation and offering evidence for early emotional monitoring and community‐based mental‐health interventions in China.

Despite increasing use of sentiment analysis in mental health research, most existing studies focus on depression, generalized anxiety, or broad negative affect. Limited evidence has examined how multiple discrete emotions co‐occur within anxiety‐ or phobia‐related discourse on Chinese social media, particularly from a nursing and psychosocial care perspective. In addition, little is known about how digital emotional expression may inform early emotional assessment, psychosocial support, stigma reduction, and community‐based mental health interventions.

Therefore, this study aims to (1) quantify the distribution of six basic emotions in phobia‐related Chinese social media comments, (2) compare emotional expression across demographic variables where data are complete, and (3) propose a preliminary model of digital affective resilience based on observed emotional co‐occurrence patterns.

## 2 Methods

This study employed a cross‐sectional observational design using secondary analysis of publicly available social media comments related to anxiety/phobia discussions.

### 2.1 Data Collection and Selection

Data were collected from publicly accessible posts and comments on XiaoHongShu, Zhihu, and Weibo between platform inception and November 1, 2025. The Chinese‐language keyword Jiaolv (anxiety) was used to collect text‐based data (posts and comments), using Python libraries (Selenium and Numpy). Only original comments (not retweets) were included. Comments shorter than five Chinese characters or consisting solely of emojis were excluded (Zhou, Lei, et al. 2025). Cross‐platform duplicates were not removed because identical user identifiers do not exist across platforms. All data analysed in this study were publicly available and anonymized prior to analysis. No identifiable personal information was collected or stored. According to institutional guidelines for non‐interventional research using publicly accessible online data, formal ethical approval and informed consent were waived. The study was conducted in accordance with relevant ethical standards for digital research and data privacy protection.

### 2.2 Text Preprocessing

To enhance model performance and reduce data noise, a seven‐step text cleaning process was implemented using Python's Numpy library: (1) Deduplication of records; (2) Handling of special characters and HTML tags; (3) Replacement of missing time and individual information with “NULL”; (4) Lowercase conversion for processing efficiency; (5) A random sample of 500 truncated comments (approximately 1.8% of the corpus) was manually reviewed by two researchers to confirm that emotion‐bearing content and contextual meaning were retained after truncation. Disagreements were resolved through discussion and consensus; (6) Text tokenization to divide text into meaningful words for analysis and (7) Removal of common but inconsequential words using a customized stop‐words list.

### 2.3 Feature Engineering

The textual data was transformed into comparable numerical features using the Term Frequency‐Inverse Document Frequency (TF‐IDF) algorithm. This step involved feature extraction, matrix construction, calculation of TF‐IDF, and normalization. The process was executed using the TfidfVectorizer component from Python's Scikit‐learn (Sklearn) library (Zhou, Qian, et al. 2025).

### 2.4 Sentiment Analysis and Validation

Several sources of bias were considered. Selection bias may arise because social media users are not representative of the general population. Information bias may result from incomplete demographic profiles and potential misclassification of emotional content by the lexicon‐based algorithm. To mitigate classification bias, manual validation was conducted on a random subsample of comments. (1) Select a lexicon: We used Dalian University of Technology's emotion dictionary, which contains word lexical categories, emotion categories, emotion intensity, and polarity. Using Ekman's emotion classification approach, the lexicon has been updated with Chinese adaptations (Zhou et al. 2024). (2) Lexical matching: The cleaned text matches the sentiment lexicon. Second, the emotion words in the text are identified. Finally, the words are labelled positive, negative, or neutral emotions. (3) Score each token: Using weighting and counting methods to score the emotion on the labelled emotion words. 4. Verification results: two independent coders manually labelled a random subsample of 1000 comments for the presence of the six emotion categories. Inter‐rater reliability was strong (Cohen's Kappa = 0.82). The sentiment classifier achieved an overall F1 score of 0.72, indicating moderate classification performance. Precision ranged from 0.65 (disgust) to 0.78 (happiness), whereas recall ranged from 0.60 (disgust) to 0.80 (happiness).

### 2.5 Statistical Analysis

Primary outcome variables were the six emotion categories identified through lexicon‐based sentiment analysis (happiness, sadness, fear, anger, surprise, and disgust). Demographic variables included gender, occupation, and education level when available from user profiles. Descriptive statistics were used to summarize the demographic variables (*n*, %) of the sample. Missing demographic data were not imputed; therefore, all demographic comparisons were based on complete‐case analysis, which may introduce bias due to non‐random missingness. To compare the differences in emotion scores across groups: The Independent Samples *t*‐test was used for gender. One‐way ANOVA was used for education level and occupation type. If a significant difference was found (*p* < 0.05), the Tukey HSD post hoc test was applied to determine the direction of the difference. All statistical analyses were conducted using SPSS 26.0 software, with the level of significance alpha = 0.05.

## 3 Results

### 3.1 Descriptive and Demographic Characteristics

A total of 28,349 valid social media comments related to phobia discussions were included in the analysis. Demographic information was partially available, with substantial proportions of missing data across variables (gender: 54.69% NULL; occupation: 51.17% NULL; education: 60.69% NULL). Therefore, all demographic comparisons were conducted using complete‐case analysis, and the effective sample size varies by variable (Table 1).

**TABLE 1:** The result of demographic characteristics.

Category	Sub‐category	Frequency (*n*)	Percentage (%)
Gender	Male	5172	18.24
	Female	7673	27.07
	NULL	15,504	54.69
	Subtotal	12,845	45.31
Occupation	Health‐related	3702	13.06
	Education/research	1416	4.99
	Other occupations	8727	30.78
	NULL	14,504	51.17
	Subtotal	13,845	48.83
Education level	High school and below	1489	5.25
	College/bachelor	8645	30.49
	Postgraduate and above	1011	3.57
	NULL	17,204	60.69
	Subtotal	11,145	39.31
Total		28,349	100.00

Among the 12,845 records with non‐missing gender data, female users accounted for 59.7% (7673/12,845), indicating a slight overrepresentation relative to male users (40.3%). Independent samples *t*‐tests based on complete cases showed that female users used significantly more happiness‐related and sadness‐related words than male users (t(12843) = 2.87, *p* = 0.005; t(12843) = 2.15, *p* = 0.034), suggesting greater emotional expressiveness in phobia‐related discourse.

For occupation, analyses were based on 13,845 complete cases. Significant differences were observed in the distribution of fear and anger expressions across occupational groups (*F* = 6.27, *p* < 0.001). Users with health‐related or psychological backgrounds showed a higher frequency of rational and regulation‐oriented terms (e.g., calmness, understanding) compared to other occupational groups (post hoc Tukey test, *p* < 0.01), indicating a potential influence of professional training on emotional framing.

For education level, 11,145 complete cases were analysed. Given the ordinal nature of the education variable, Spearman's rank correlation was used. A significant positive association was observed between education level and anger‐related expression (ρ = 0.33, *p* < 0.01). However, this finding should be interpreted cautiously due to the high proportion of missing data (60.69%) and its exploratory nature.

Table 1 presents the full distribution of demographic characteristics. Percentages are calculated based on the total sample (*N* = 28,349), while subtotals indicate the number of complete cases available for each variable.

### 3.2 Distribution of Sentiment Analysis

Emotion percentages refer to the proportion of emotion‐labelled words identified in the corpus rather than the proportion of comments containing at least one emotion word. Happiness (31.6%) and Surprise (15.3%) were the most frequent emotions observed, followed by Fear (18.4%), Sadness (14.7%), Anger (12.1%), and Disgust (7.9%) (Table 2).

**TABLE 2:** Sentiment analysis results: Emotion category, representative keywords, and descriptive word list.

Emotion	Related sentiment analysis word	Sentiment analysis theme
Sad	Empathy, compassion, care, pity, understand, sympathy, grieve, miss, pray	Empathy as an emotional bridge
Anger	Reason, standard, authority, frustration, criticism, dispute, demand, rational, justice	Rationalized emotion and epistemic control
Fear	Trust, calmness, courage, brave, anxious, terrified, safe, avoid, panic, reappraise	Cognitive reappraisal and exposure through discourse
Disgust	Acceptance, normal, integrate, stigma, shame, exclusion, tolerance, openness	Destigmatization and emotional integration
Surprise	Discover, interesting, inspire, curiosity, explore, learn, novel, awe, insight	Cognitive expansion and digital health literacy
Happy	Humour, support, resilience, optimism, gratitude, joy, connect, shared, relieve	The social antidote to anxiety

## 4 Discussion

This study examined affective expressions in social media discussions related to phobia and identified a heterogeneous emotional profile rather than a predominantly fear‐centred pattern. Although fear‐related content accounted for 18.4% of emotion‐labelled words, it was not the most prevalent category, with happiness (31.6%) and surprise (15.3%) occurring more frequently. This distribution indicates that phobia‐related discourse in this dataset includes a range of emotional expressions beyond fear, such as supportive, empathetic, and curiosity‐oriented language. However, relative to studies of depression discourse on social media, where negative affect often exceeds 50% (Ayed et al. 2025), the 18.4% fear observed here is notably lower. Therefore, these findings should be interpreted cautiously, and no conclusions can be drawn regarding how this pattern differs from other forms of mental health–related discourse.

### 4.1 From Individual Emotion to Collective Regulation

Traditional psychological models conceptualize fear as a self‐protective emotion that triggers withdrawal or avoidance (Dunsmoor and Murphy 2015). However, the current findings reveal that within online communities, fear is not merely expressed but collectively reframed. In the discourse, fear was linguistically framed as a communal experience through shared narratives and symbolic interaction, consistent with social regulation theory of emotion. This supports the social regulation theory of emotion (Hogeveen et al. 2016), which posits that emotional experiences are co‐constructed through interpersonal processes rather than isolated internal states (Yang et al. 2021).

Moreover, the data indicate that emotion in digital discourse follows a trajectory rather than a static polarity. The co‐occurrence of fear‐related, curiosity‐oriented, and empathy‐related language suggests that emotional expression within digital discourse is multidimensional rather than limited to isolated negative affect (Yang et al. 2020). This aligns with the emotion regulation framework, particularly the mechanism of cognitive reappraisal, where individuals reinterpret stressors to regain a sense of control. In clinical terms, this transformation mirrors adaptive coping observed in cognitive‐behavioural therapy. The observed linguistic patterns are consistent with the idea that online discourse may serve supportive functions; however, the present study does not directly measure therapeutic outcomes or emotional improvement.

### 4.2 Emotional Pathways and Their Clinical Significance

#### 4.2.1 Happiness—The Social Antidote to Anxiety

Happiness emerged as the most frequent emotional category, often intertwined with expressions of empathy, humour, and mutual support. Positive affect in this context performs a reparative social function, transforming emotional vulnerability into a shared experience of resilience. This finding is consistent with Fredrickson's broaden‐and‐build theory (Fredrickson 2004), suggesting that positive emotions may broaden cognitive scope and foster adaptive psychological resources (Aqtam, Ayed, et al. 2025). Clinically, this pattern underscores the importance of positive affect priming in psychological interventions (Coventry et al. 2020; Förster and Kanske 2022). Digital environments that facilitate humour, gratitude expression, or peer appreciation may enhance engagement and emotional recovery. For instance, integrating cues into AI chatbots or online counselling platforms could foster an affective climate that buffers anxiety and improves emotional regulation among patients with phobic symptoms (Etkin and Wager 2007).

#### 4.2.2 Sadness—Empathy as an Emotional Bridge

Rather than signifying helplessness, sadness in this dataset served as an empathic connector. The use of compassionate language (“care,” “pray,” “miss,” “understand”) suggests that sadness functions as a form of moral resonance. This supports research indicating that sadness in collectivist cultures can enhance prosocial behaviour and communal cohesion. The results of this study are consistent with previous studies (Elliott et al. 2011; Christov‐Moore et al. 2014). In the healthcare setting, recognizing sadness as an adaptive social emotion is critical. When patients or family members express sadness online, it may signal a desire for emotional validation rather than a pathological depressive state (Gordon et al. 2020). Accordingly, training nurses and mental health professionals to interpret sadness through a compassion‐based lens can improve relational empathy and strengthen the therapeutic alliance (Pinto et al. 2012).

#### 4.2.3 Fear—Cognitive Reappraisal and Exposure Through Discourse

Fear is a central component of phobia‐related discourse; however, in this dataset it accounted for 18.4% of emotion‐labelled words and was not the most prevalent category. Notably, fear‐related expressions frequently co‐occurred with terms such as calmness, courage and understanding, suggesting that users often frame fear within a broader context of coping‐oriented language. This pattern indicates that discussions of fear are not limited to expressions of distress but may also include elements of reflection and reinterpretation (Craske et al. 2014). The co‐occurrence of fear‐related terms with reappraisal‐related words, such as calmness and courage, is conceptually consistent with previous neurobiological studies of fear regulation and cognitive reappraisal (Izquierdo et al. 2016; Garcia 2017). From a theoretical perspective, such co‐occurrence is consistent with the concept of cognitive reappraisal, a key mechanism in cognitive‐behavioural frameworks, in which individuals reinterpret threatening stimuli to reduce emotional impact. However, this interpretation should be made cautiously (Paquet et al. 2023). The present analysis is based on cross‐sectional, aggregate text data and does not capture individual‐level emotional trajectories or causal processes. Therefore, while the observed linguistic patterns are conceptually aligned with reappraisal, they should not be interpreted as direct evidence of therapeutic processes such as exposure or clinical improvement. Taken together, these findings suggest that fear‐related discourse in online settings may incorporate both distress expression and coping‐oriented language, reflecting a complex and socially mediated pattern of emotional communication.

#### 4.2.4 Anger—Rationalized Emotion and Epistemic Control

Anger‐related expressions appeared rational and reflective, emphasizing reason, standards, and authority. This pattern is consistent with Constructive Anger Theory, although it may also reflect Chinese online discourse norms that favour rationalized rather than openly hostile expression (Adams 2019). The results of this study are consistent with previous studies (Botha 2021). In patient–clinician interactions, this finding suggests that expressions of frustration may signal informational or epistemic gaps rather than interpersonal hostility. Healthcare professionals who recognize this dimension can respond with transparency and shared decision‐making, converting potential conflict into engagement. This finding extends the literature on emotional labor and communication competence in nursing, emphasizing that appropriate recognition of rationalized anger may reduce misunderstandings and improve patient satisfaction.

#### 4.2.5 Surprise—Cognitive Expansion and Digital Health Literacy

Surprise‐related expressions (“discover,” “interesting,” “inspire”) indicate curiosity rather than shock, revealing a shift from emotional avoidance to epistemic engagement. This transformation aligns with Berlyne's Arousal Theory of Curiosity, where moderate uncertainty stimulates exploration and learning (Kidd and Hayden 2015). The results of this study are consistent with previous studies (Kaanders et al. 2021). In medical communication, surprise plays a unique role: it activates cognitive openness and facilitates health information seeking (MoshirPanahi et al. 2020). Thus, emotional novelty—when managed ethically—can enhance digital health literacy. For example, health education tools that introduce mildly surprising facts or narratives can increase user engagement and promote reflective understanding rather than anxiety‐driven avoidance, defence of autonomy and cognitive control (Looyestyn et al. 2017).

#### 4.2.6 Disgust—Destigmatization and Emotional Integration

Disgust was the least frequent emotion category, but it was still present. Its lower frequency may reflect cultural patterns of expression in Chinese mental‐health discourse, rather than the disappearance of stigma alone. Traditionally, disgust functions as a social boundary emotion distinguishing “normal” from “deviant.” Its absence suggests that psychological vulnerability is no longer stigmatized but integrated into collective empathy. This reflects a shift from a shame culture to what may be termed an acceptance culture, where open emotional expression is morally legitimate. This study first verifies the results. This pattern may reflect a shift from shame‐oriented stigma toward greater emotional acceptance within online discourse. Clinically, this transformation holds important implications for mental health stigma reduction (Saucier 2018). Digital spaces that normalize fear, anxiety, and vulnerability may encourage individuals to seek help earlier and engage more openly with healthcare providers (Yogarajah et al. 2020). This supports ongoing global efforts to use social media as a platform for mental health destigmatization (Whiteman et al. 2016).

### 4.3 Integrative Emotional Dynamics and Theoretical Synthesis

Across the dataset, the six emotion categories do not appear in isolation but show systematic patterns of co‐occurrence within phobia‐related discourse. In particular, fear‐related expressions are frequently accompanied by empathy‐related language (e.g., sadness), supportive or positive expressions (happiness), and, in some cases, rational or evaluative terms (anger) and curiosity‐oriented language (surprise). Disgust‐related expressions were comparatively infrequent but present. These patterns indicate that emotional expression in this context is multidimensional rather than dominated by a single affective state.

Based on these observations, we propose a conceptual model of digital affective dynamics in which fear is embedded within a broader network of co‐occurring emotional and cognitive expressions. Importantly, this model does not represent a temporal sequence or developmental pathway (Zhou et al. 2023). The data are cross‐sectional and aggregated at the corpus level; therefore, no conclusions can be drawn about transitions between emotional states at the individual level.

The findings can be interpreted through two complementary theoretical perspectives. First, social regulation of emotion provides a framework for understanding how emotional expressions are shaped through interaction in online environments, where users respond to and reinterpret each other's experiences (Peng et al. 2024). Second, the frequent co‐occurrence of fear‐related terms is consistent with the concept of cognitive reappraisal, in which individuals reinterpret threatening experiences to reduce emotional impact (Aqtam, Shouli, et al. 2025; Aqtam et al. 2023). These frameworks together suggest that emotional expression in social media discourse reflects not only individual affect but also socially mediated processes of interpretation (Szeto et al. 2024). However, these interpretations remain tentative, as the present analysis is based on cross‐sectional text data and does not directly measure underlying psychological mechanisms.

### 4.4 Implications for Practice and Policy

Healthcare professionals should interpret online emotional expression as potential signals of coping, adaptation, and psychosocial needs rather than merely isolated emotional states. The coexistence of fear with supportive, empathetic, and coping‐oriented language suggests that digital platforms may function as spaces for emotional disclosure, collective support, and cognitive reappraisal (Yang and Kim 2022; Shen et al. 2024). Training in affective literacy and digital empathy could help nurses engage more effectively with individuals expressing emotional distress through social media or AI‐based communication platforms (Farahani et al. 2024). In addition, positive emotion cultivation strategies, such as humour, gratitude expression, and peer support, may complement conventional anxiety management approaches (Ahmed et al. 2024).

The relatively low frequency of disgust‐related expressions may reflect a gradual reduction in mental health stigma within online discourse, suggesting that ethically guided social media environments can promote supportive health communication and emotional acceptance (Wu et al. 2025; Rasool and Hassan 2025). These findings highlight the emotional interdependence between individuals and their social environment and emphasize the potential value of community‐based digital mental health interventions (Zhang et al. 2025; Nazari et al. 2023). Family‐centered psychoeducation, online support groups, and AI‐assisted emotional monitoring systems may help strengthen early emotional support, mental health literacy, and patient‐centered psychosocial care in nursing practice (Obieche et al. 2025).

### 4.5 Limitations and Future Directions

Several limitations should be considered when interpreting the findings of this study. First, substantial demographic missingness (gender 54.69%, occupation 51.17%, education 60.69%) limited subgroup analyses. All demographic comparisons were based on complete‐case analysis without imputation, which may introduce bias due to non‐random missingness. Second, the sentiment classifier achieved moderate performance (F1 = 0.72). Although inter‐rater reliability was strong (Cohen's Kappa = 0.82), lexicon‐based sentiment analysis may misclassify context‐dependent, ironic, or culturally nuanced expressions. In addition, the Dalian University of Technology emotion dictionary may not fully capture emerging internet slang or platform‐specific emotional language. Third, the study relied on convenience sampling from XiaoHongShu, Zhihu and Weibo, limiting generalizability to other platforms or cultural contexts. Platform recommendation algorithms may also have overrepresented emotionally engaging or highly visible content. Fourth, the emotional expressions analysed were not validated against clinical diagnoses of anxiety or phobia. Therefore, the findings reflect subjective online discourse rather than clinically confirmed conditions. Fifth, the study used cross‐sectional aggregate text data and did not track users longitudinally. As a result, interpretations regarding emotional regulation or adaptation are inferred from co‐occurrence patterns rather than directly observed psychological change. Finally, no qualitative thematic analysis was conducted to complement the quantitative sentiment analysis. Future research should incorporate longitudinal designs, mixed methods, multimodal data, and clinical validation to better understand the relationship between online emotional expression and real‐world mental health outcomes.

## 5 Conclusion

This study shows that phobia‐related discourse on Chinese social media reflects co‐occurring emotional expressions rather than fear alone. These patterns suggest that online communication may play a role in shaping how anxiety is expressed and interpreted. These patterns suggest that online communication may play a role in shaping how anxiety is expressed and interpreted. Future research should incorporate longitudinal designs, clinical validation, and multimodal data (e.g., text, images, and physiological signals) to determine whether observed co‐occurrence patterns reflect genuine emotional regulation processes or broader discursive norms. From a practical perspective, AI‐based moderation or conversational systems could be designed to detect and prioritize supportive and empathetic content, although such applications require ethical safeguards and further validation.

## Author Contributions

Jin G., Z.W., L.Y., Y.Y., Q.X., S.Z., X.T. and L.Y. contributed to the conceptualization, and writing – original draft. Jinji C. and Q.Z. contributed to the conceptualization, data curation, formal analysis, funding acquisition, investigation, methodology, project administration, software, resources, supervision, validation, visualization, writing – original draft, writing – review and editing.

## Funding

The authors did not receive any specific funding for this study.

## Ethics Statement

The study did not involve human subjects, and the data came from publicly available platforms for academic research. Therefore, after the Institutional Review Board's approval, the researchers only collected comments, which did not involve human subjects' privacy and did not cause human subjects' harm.

## Consent

The authors have nothing to report.

## Conflicts of Interest

The authors declare no conflicts of interest.

## Data Availability

The data that support the findings of this study are available from the corresponding author upon reasonable request.

## References

[nop270691-bib-0001] Adams, C. D. 2019. “A Brief Tour of Epidemiologic Epigenetics and Mental Health.” Current Opinion in Psychology 27: 36–40. 10.1016/j.copsyc.2018.07.010. 30121470

[nop270691-bib-0002] Ahmed, O. , E. I. Walsh , A. Dawel , K. Alateeq , D. A. Espinoza Oyarce , and N. Cherbuin . 2024. “Social Media Use, Mental Health and Sleep: A Systematic Review With Meta‐Analyses.” Journal of Affective Disorders 367: 701–712. 10.1016/j.jad.2024.08.193. 39242043

[nop270691-bib-0003] Apicella, A. , P. Arpaia , S. Barbato , et al. 2025. “Domain Adaptation for Fear of Heights Classification in a VR Environment Based on EEG and ECG.” Information Systems Frontiers 27: 139–154. 10.1007/s10796-024-10484-z.

[nop270691-bib-0004] Aqtam, I. 2025. “A Narrative Review of Mental Health and Psychosocial Impact of the War in Gaza.” East Mediterranean Health Journal 31, no. 2: 89–96. 10.26719/2025.31.2.89. 40116261

[nop270691-bib-0005] Aqtam, I. , A. Ayed , O. A. Alfuqaha , and M. Shouli . 2025. “Stress and Resilience of Nursing Students in Clinical Training During Political Violence: A Palestinian Perspective.” PLoS One 20, no. 6: e0325278. 10.1371/journal.pone.0325278. 40560858 PMC12192045

[nop270691-bib-0006] Aqtam, I. , A. Ayed , D. Toqan , et al. 2023. “The Relationship Between Stress and Resilience of Nurses in Intensive Care Units During the COVID‐19 Pandemic.” Inquiry 60: 00469580231179876. 10.1177/00469580231179876. PMC10247682 37278278

[nop270691-bib-0007] Aqtam, I. , R. Naghnaghiyeh , T. Zubaide , Y. Othman , and Y. Malasah . 2025. “Psychological Impact of Political Conflict: Prevalence and Severity of PTSD Among Children and Adolescents in the North and Middle West Bank Following the October 7, 2023, Events.” Nursing Research and Practice 2025: 7304673. 10.1155/nrp/7304673. 40810006 PMC12349999

[nop270691-bib-0008] Aqtam, I. , M. Shouli , S. Alqoroum , and K. Shouli . 2025. “Mental Health Interventions for Resilience and Mental Health in Conflict‐Affected Palestinian Communities: A Systematic Review Revealing Research Gaps and the Absence of Narrative Therapy Evidence.” Global Mental Health (Cambridge) 12: e143. 10.1017/gmh.2025.10103. PMC12720377 41438269

[nop270691-bib-0009] Ayed, A. , M. A. Ejheisheh , R. Al‐Amer , et al. 2025. “Insights Into the Relationship Between Anxiety and Attitudes Toward Artificial Intelligence Among Nursing Students.” BMC Nursing 24: 812. 10.1186/s12912-025-03490-2. 40597186 PMC12211391

[nop270691-bib-0010] Botha, M. 2021. “Academic, Activist, or Advocate? Angry, Entangled, and Emerging: A Critical Reflection on Autism Knowledge Production.” Frontiers in Psychology 12: 727542. 10.3389/fpsyg.2021.727542. 34650484 PMC8506216

[nop270691-bib-0011] Christov‐Moore, L. , E. A. Simpson , G. Coudé , K. Grigaityte , M. Iacoboni , and P. F. Ferrari . 2014. “Empathy: Gender Effects in Brain and Behavior.” Neuroscience and Biobehavioral Reviews 46, no. Pt 4: 604–627. 10.1016/j.neubiorev.2014.09.001. 25236781 PMC5110041

[nop270691-bib-0012] Coventry, P. A. , N. Meader , H. Melton , et al. 2020. “Psychological and Pharmacological Interventions for Posttraumatic Stress Disorder and Comorbid Mental Health Problems Following Complex Traumatic Events: Systematic Review and Component Network Meta‐Analysis.” PLoS Medicine 17: e1003262. 10.1371/journal.pmed.1003262. 32813696 PMC7446790

[nop270691-bib-0013] Craske, M. G. , A. N. Niles , L. J. Burklund , et al. 2014. “Randomized Controlled Trial of Cognitive Behavioral Therapy and Acceptance and Commitment Therapy for Social Phobia: Outcomes and Moderators.” Journal of Consulting and Clinical Psychology 82: 1034–1048. 10.1037/a0037212. 24999670 PMC4244236

[nop270691-bib-0014] Crocq, M.‐A. 2015. “A History of Anxiety: From Hippocrates to DSM.” Dialogues in Clinical Neuroscience 17: 319–325. 10.31887/DCNS.2015.17.3/macrocq. 26487812 PMC4610616

[nop270691-bib-0015] Dunsmoor, J. E. , and G. L. Murphy . 2015. “Categories, Concepts, and Conditioning: How Humans Generalize Fear.” Trends in Cognitive Sciences 19: 73–77. 10.1016/j.tics.2014.12.003. 25577706 PMC4318701

[nop270691-bib-0016] Elliott, R. , A. C. Bohart , J. C. Watson , and L. S. Greenberg . 2011. “Empathy.” Psychotherapy (Chicago, Ill.) 48: 43–49. 10.1037/a0022187. 21401273

[nop270691-bib-0017] Etkin, A. , and T. D. Wager . 2007. “Functional Neuroimaging of Anxiety: A Meta‐Analysis of Emotional Processing in PTSD, Social Anxiety Disorder, and Specific Phobia.” American Journal of Psychiatry 164: 1476–1488. 10.1176/appi.ajp.2007.07030504. 17898336 PMC3318959

[nop270691-bib-0018] Farahani, M. A. , S. Nargesi , N. Saniee , Z. Dolatshahi , F. Heidari Beni , and S. Shariatpanahi . 2024. “Factors Affecting Nurses Retention During the COVID‐19 Pandemic: A Systematic Review.” Human Resources for Health 22: 78. 10.1186/s12960-024-00960-7. 39567984 PMC11580645

[nop270691-bib-0019] Förster, K. , and P. Kanske . 2022. “Upregulating Positive Affect Through Compassion: Psychological and Physiological Evidence.” International Journal of Psychophysiology 176: 100–107. 10.1016/j.ijpsycho.2022.03.009. 35358613

[nop270691-bib-0020] Fredrickson, B. L. 2004. “The Broaden‐And‐Build Theory of Positive Emotions.” Philosophical Transactions of the Royal Society of London. Series B, Biological Sciences 359: 1367–1378. 10.1098/rstb.2004.1512. 15347528 PMC1693418

[nop270691-bib-0021] Garcia, R. 2017. “Neurobiology of Fear and Specific Phobias.” Learning & Memory 24: 462–471. 10.1101/lm.044115.116. 28814472 PMC5580526

[nop270691-bib-0022] Glaz, A. L. , Y. Haralambous , D.‐H. Kim‐Dufor , et al. 2021. “Machine Learning and Natural Language Processing in Mental Health: Systematic Review.” Journal of Medical Internet Research 23: e15708. 10.2196/15708. 33944788 PMC8132982

[nop270691-bib-0023] Gordon, B. R. , C. P. McDowell , M. Lyons , and M. P. Herring . 2020. “Resistance Exercise Training for Anxiety and Worry Symptoms Among Young Adults: A Randomized Controlled Trial.” Scientific Reports 10: 17548. 10.1038/s41598-020-74608-6. 33067493 PMC7567848

[nop270691-bib-0024] Hogeveen, J. , C. Salvi , and J. Grafman . 2016. ““Emotional Intelligence”: Lessons From Lesions.” Trends in Neurosciences 39: 694–705. 10.1016/j.tins.2016.08.007. 27647325 PMC5807001

[nop270691-bib-0025] Izquierdo, I. , C. R. G. Furini , and J. C. Myskiw . 2016. “Fear Memory.” Physiological Reviews 96: 695–750. 10.1152/physrev.00018.2015. 26983799

[nop270691-bib-0026] Julian, L. J. 2011. “Measures of Anxiety: State‐Trait Anxiety Inventory (STAI), Beck Anxiety Inventory (BAI), and Hospital Anxiety and Depression Scale‐Anxiety (HADS‐A).” Arthritis Care & Research (Hoboken, N.J.) 63, no. Suppl 11 0 11: S467–S472. 10.1002/acr.20561. PMC3879951 22588767

[nop270691-bib-0027] Kaanders, P. , K. Juechems , J. O'Reilly , and L. Hunt . 2021. “Dissociable Mechanisms of Information Sampling in Prefrontal Cortex and the Dopaminergic System.” Current Opinion in Behavioral Sciences 41: 63–70. 10.1016/j.cobeha.2021.04.005.

[nop270691-bib-0028] Kidd, C. , and B. Y. Hayden . 2015. “The Psychology and Neuroscience of Curiosity.” Neuron 88: 449–460. 10.1016/j.neuron.2015.09.010. 26539887 PMC4635443

[nop270691-bib-0029] Koskinen, M.‐K. , and I. Hovatta . 2023. “Genetic Insights Into the Neurobiology of Anxiety.” Trends in Neurosciences 46: 318–331. 10.1016/j.tins.2023.01.007. 36828693

[nop270691-bib-0030] Looyestyn, J. , J. Kernot , K. Boshoff , J. Ryan , S. Edney , and C. Maher . 2017. “Does Gamification Increase Engagement With Online Programs? A Systematic Review.” PLoS One 12: e0173403. 10.1371/journal.pone.0173403. 28362821 PMC5376078

[nop270691-bib-0031] MoshirPanahi, S. , A. R. Moradi , B. Ghaderi , C. McEwen , and L. Jobson . 2020. “Predictors of Positive and Negative Post‐Traumatic Psychological Outcomes in a Sample of Iranian Cancer Survivors.” British Journal of Health Psychology 25: 390–404. 10.1111/bjhp.12412. 32348016

[nop270691-bib-0032] Nazari, A. , G. Garmaroudi , A. R. Foroushani , and M. Hosseinnia . 2023. “The Effect of Web‐Based Educational Interventions on Mental Health Literacy, Stigma and Help‐Seeking Intentions/Attitudes in Young People: Systematic Review and Meta‐Analysis.” BMC Psychiatry 23: 647. 10.1186/s12888-023-05143-7. 37667229 PMC10478184

[nop270691-bib-0033] Obieche, O. , J.‐Y. Tan , S. Sharma , D. Bressington , and T. Wang . 2025. “Telehealth Family Psychoeducation for Major Depressive Disorder: A Protocol for Intervention co‐Design and Feasibility Study.” Nursing Reports 15: 364. 10.3390/nursrep15100364. 41149679 PMC12566970

[nop270691-bib-0034] Paquet, C. , J. Whitehead , R. Shah , et al. 2023. “Social Prescription Interventions Addressing Social Isolation and Loneliness in Older Adults: Meta‐Review Integrating On‐The‐Ground Resources.” Journal of Medical Internet Research 25: e40213. 10.2196/40213. 37195738 PMC10233446

[nop270691-bib-0035] Peng, L. , J. Wang , N. Zheng , and X. Guo . 2024. “Traversing Emotional Spaces: Social Media Affordances and Emotion Regulation in Times of Physical Isolation.” Social Media + Society 10: 20563051241237273. 10.1177/20563051241237273.

[nop270691-bib-0036] Phiri, D. , F. Makowa , V. L. Amelia , Y. V. A. Phiri , L. P. Dlamini , and M.‐H. Chung . 2025. “Text‐Based Depression Prediction on Social Media Using Machine Learning: Systematic Review and Meta‐Analysis.” Journal of Medical Internet Research 27: e59002. 10.2196/59002. 40215481 PMC12032503

[nop270691-bib-0037] Pinto, R. Z. , M. L. Ferreira , V. C. Oliveira , et al. 2012. “Patient‐Centred Communication Is Associated With Positive Therapeutic Alliance: A Systematic Review.” Journal of Physiotherapy 58: 77–87. 10.1016/S1836-9553(12)70087-5. 22613237

[nop270691-bib-0038] Pitman, A. , S. Suleman , N. Hyde , and A. Hodgkiss . 2018. “Depression and Anxiety in Patients With Cancer.” BMJ 361: k1415. 10.1136/bmj.k1415. 29695476

[nop270691-bib-0039] Rasool, N. , and M. Hassan . 2025. “The Role of Digital Mental Healthcare in Reducing Mental Health Stigma: Insights From the Covid‐ 19 Pandemic.” Current Psychology 44: 14138–14150. 10.1007/s12144-025-07803-1.

[nop270691-bib-0040] Russo, S. , I. E. Tibermacine , A. Tibermacine , et al. 2024. “Analyzing EEG Patterns in Young Adults Exposed to Different Acrophobia Levels: A VR Study.” Frontiers in Human Neuroscience 18: 18. 10.3389/fnhum.2024.1348154. PMC11102978 38770396

[nop270691-bib-0041] Saeed, S. A. , K. Cunningham , and R. M. Bloch . 2019. “Depression and Anxiety Disorders: Benefits of Exercise, Yoga, and Meditation.” American Family Physician 99: 620–627. 31083878

[nop270691-bib-0042] Saucier, G. 2018. “Culture, Morality and Individual Differences: Comparability and Incomparability Across Species.” Philosophical Transactions of the Royal Society, B: Biological Sciences 373: 20170170. 10.1098/rstb.2017.0170. PMC5832693 29483353

[nop270691-bib-0043] Shen, H. , C. Chen , S. Yan , et al. 2024. “Online Digital Health and Informatics Education for Undergraduate Nursing Students in China: Impacts and Recommendations.” BMC Medical Education 24: 803. 10.1186/s12909-024-05785-5. 39061003 PMC11282779

[nop270691-bib-0044] Song, J. , M. Wang , and Y. Li . 2024. “An Exploratory Study of Deep Learning‐Based Sentiment Analysis Among Weibo Users in China.” Current Psychology 43: 15213–15226. 10.1007/s12144-023-05493-1.

[nop270691-bib-0045] Szeto, S. , A. K. Y. Au , and S. K. L. Cheng . 2024. “Support From Social Media During the COVID‐19 Pandemic: A Systematic Review.” Behavioral Science 14: 759. 10.3390/bs14090759. PMC11428965 39335974

[nop270691-bib-0046] Whiteman, K. L. , J. A. Naslund , E. A. DiNapoli , M. L. Bruce , and S. J. Bartels . 2016. “Systematic Review of Integrated General Medical and Psychiatric Self‐Management Interventions for Adults With Serious Mental Illness.” Psychiatric Services 67: 1213–1225. 10.1176/appi.ps.201500521. 27301767 PMC5089924

[nop270691-bib-0047] Wu, P. , S. Zou , C. Chen , and Y. Song . 2025. “Hotbed of Stigmatization or Source of Support: A Multimodal Analysis of Mental Health‐Related Videos on Douyin.” Computers in Human Behavior 172: 108716. 10.1016/j.chb.2025.108716.

[nop270691-bib-0048] Yang, J. , and S. Kim . 2022. “An Online Communication Skills Training Program for Nursing Students: A Quasi‐Experimental Study.” PLoS One 17: e0268016. 10.1371/journal.pone.0268016. 35507577 PMC9067637

[nop270691-bib-0049] Yang, Y. , K. Liu , S. Li , and M. Shu . 2020. “Social Media Activities, Emotion Regulation Strategies, and Their Interactions on People's Mental Health in COVID‐19 Pandemic.” International Journal of Environmental Research and Public Health 17: 8931. 10.3390/ijerph17238931. 33271779 PMC7730977

[nop270691-bib-0050] Yang, Y. , N. Ta , K. Li , F. Jiao , B. Hu , and Z. Li . 2021. “Influential Factors on Collective Anxiety of Online Topic‐Based Communities.” Frontiers in Psychology 12: 12. 10.3389/fpsyg.2021.740065. PMC8525538 34675846

[nop270691-bib-0051] Yogarajah, A. , R. Kenter , Y. Lamo , V. Kaldo , and T. Nordgreen . 2020. “Internet‐Delivered Mental Health Treatment Systems in Scandinavia – A Usability Evaluation.” Internet Interventions 20: 100314. 10.1016/j.invent.2020.100314. 32426241 PMC7226875

[nop270691-bib-0052] Zhang, Z. , N. Reavley , G. Armstrong , and A. Morgan . 2025. “Public Disclosures of Mental Health Problems on Social Media and Audiences' Self‐Reported Anti‐Stigma Effects.” Health Promotion International 40: daae204. 10.1093/heapro/daae204. 39835579 PMC11747871

[nop270691-bib-0053] Zhou, Q. , Y. Lei , L. Tian , S. Ai , Y. Yang , and Y. Zhu . 2025. “Perception and Sentiment Analysis of Palliative Care in Chinese Social Media: Qualitative Studies Based on Machine Learning.” Social Science & Medicine 379: 118178. 10.1016/j.socscimed.2025.118178. 40381285

[nop270691-bib-0054] Zhou, Q. , L. Qian , L. Wu , et al. 2025. “Sentiment Analysis of Cancer Screening in Chinese Social Media: Qualitative Studies Based on Machine Learning.” PLoS One 20: e0336119. 10.1371/journal.pone.0336119. 41191632 PMC12588461

[nop270691-bib-0055] Zhou, Q. , Y. Xu , L. Yang , and R. Menhas . 2024. “Attitudes of the Public and Medical Professionals Toward Nurse Prescribing: A Text‐Mining Study Based on Social Medias.” International Journal of Nursing Sciences 11: 99–105. 10.1016/j.ijnss.2023.12.005. 38352288 PMC10859581

[nop270691-bib-0056] Zhou, S. , X. Yang , Y. Wang , X. Zheng , and Z. Zhang . 2023. “Affective Agenda Dynamics on Social Media: Interactions of Emotional Content Posted by the Public, Government, and Media During the COVID‐19 Pandemic.” Humanities & Social Sciences Communications 10: 797. 10.1057/s41599-023-02265-x.

